# A Finite Element Study of the Dynamic Response of Brain Based on Two Parasagittal Slice Models

**DOI:** 10.1155/2015/816405

**Published:** 2015-09-30

**Authors:** Xuewei Song, Cong Wang, Hao Hu, Tianlun Huang, Jingxu Jin

**Affiliations:** State Key Laboratory of Automobile Dynamic Simulation, Jilin University, Changchun 130025, China

## Abstract

The objective of this study is to investigate the influence of gyri and sulci on the response of human head under transient loading. To this end, two detailed parasagittal slice models with and without gyri and sulci have been developed. The models comprised not only cerebrum and skull but also cerebellum, brain stem, CSF, and corpus callosum. In addition, white and gray matters were separated. The material properties were adopted from the literature and assigned to different parts of the models. Nahum's and Trosseille's experiments reported in relevant literature were simulated and the simulation results were compared with the test data. The results show that there is no evident difference in terms of intracranial pressure between the models with and without gyri and sulci under simulated conditions. The equivalent stress below gyri and sulci in the model with gyri and sulci is slightly higher than that in the counterpart model without gyri and sulci. The maximum principle strain in brain tissue is lower in the model with gyri and sulci. The stress and strain distributions are changed due to the existence of gyri and sulci. These findings highlight the necessity to include gyri and sulci in the finite element head modeling.

## 1. Introduction

Brain injury is the leading cause of morbidity and mortality in road accidents and brings a lot of social and economic problems. In the United States, about 200,000 cases of traumatic brain injury occur each year, and up to 90 to 10 billion dollars are spent for treatment [1]. In Europe, the incidence rate and mortality rate are about 235 and 15.4 per 100,000 of the population each year, respectively [2]. Similar rates have been shown in France and China [3, 4]. Due to the large amount of traffic injuries with head trauma, it is crucial to investigate the mechanisms of such injury in detail for better treatment. Generally, there are three approaches for injury studies, namely, physical tests, analytical modeling, and numerical simulations. Due to the low cost and high accuracy, numerical simulations have been widely accepted as the best way and partial alternative to the physical tests. With the help of numerical models, typically finite element (FE) model, biomechanical responses, such as intracranial pressure, stress, and strain of brain tissues, can be calculated, and the mechanism of the head traumatic brain injury can be further studied.

In spite of a three-dimensional structure of human head, two-dimensional models also can be used to study the human injury mechanisms and make an effective assessment on brain injury. In the last few decades, various finite element models for investigating head dynamic response to transient loading have been reported [5–11], and some of them are two-dimensional head models [5–7, 11]. However, gyri and sulci were usually ignored or roughly described and represented by a homogeneous geometry in these models. Gyri and sulci are formed due to the convolution of the cerebral cortex and cover the surface of the brain, which create a complex neuron network [12, 13]. Therefore, it is necessary to study the influence of gyri and sulci in head impact.

In recent studies there were also numerous efforts to investigate the influence of gyri and sulci. Bradshaw and Morfey [14] developed two-dimensional models with and without sulci to investigate the influence of the sulci on gross cerebrum motion. The results showed that the sulci had no significant effect on the displacements between skull and brain.

Cloots et al. [15] made a comparison between local parts of the cortex with different sulci geometrical profiles and a homogenous part without gyri and sulci. Two loading conditions were simulated, which resulted in different equivalent stress fields. The predicted stresses based on different geometries were also different. It was suggested that gyri and sulci should be considered in finite element models to make more accurate injury assessments.

Lauret et al. [16] measured strain fields with high resolution in the sagittal sections of brain tissue with the sulci and simulated the brain response at translational accelerations. Slices were obtained from fresh porcine brain tissue, and higher von Mises strains were found between sulci.

Ho and Kleiven [17] compared the strains using two models with and without sulci under impact simulations. It was indicated that the strain distribution in the brain tissue was altered due to sulci, which should be included in future FE models. However, it was not shown whether gyri and sulci had the same influence on intracranial pressure and stress under different impact conditions.

Although the aforementioned studies provided encouraging results for investigating the influence of gyri and sulci on head injuries, only strain or stress in a single simulated condition was observed and compared. The effect of gyri and sulci on intracranial pressure was not comprehensively investigated under different loading conditions in previous literatures. In addition, the characteristics of the gyri and sulci in previous models were not clearly described in detail.

In this paper, the dynamic response of parasagittal plane under transient loading was investigated. Two parasagittal slice finite element models with and without gyri and sulci of a parasagittal slice of a human head have been developed and then were validated using the experiment data in Nahum et al. [24] and Trosseille et al. [25]. Loading conditions were consistent with the physical tests and material parameters were based on the published literatures.

## 2. Methods

### 2.1. Model Description

**Figure 1:**
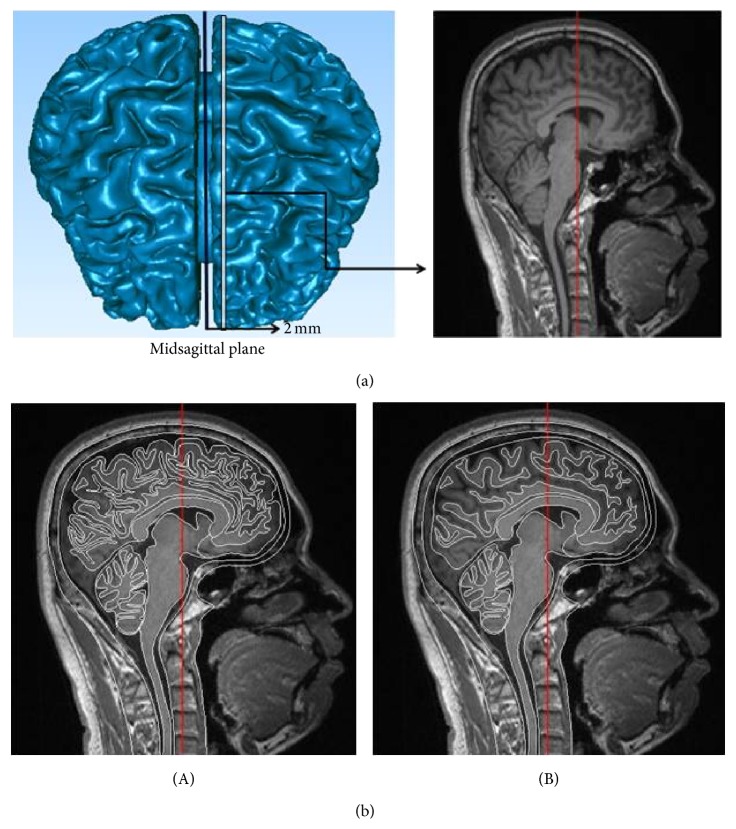
(a) The chosen slice position and parasagittal MRI data. (b) The extracted geometric profile of the chosen parasagittal MRI data using CATIA. (A) The geometric profile with gyri and sulci. (B) The geometric profile without gyri and sulci.

**Figure 2:**
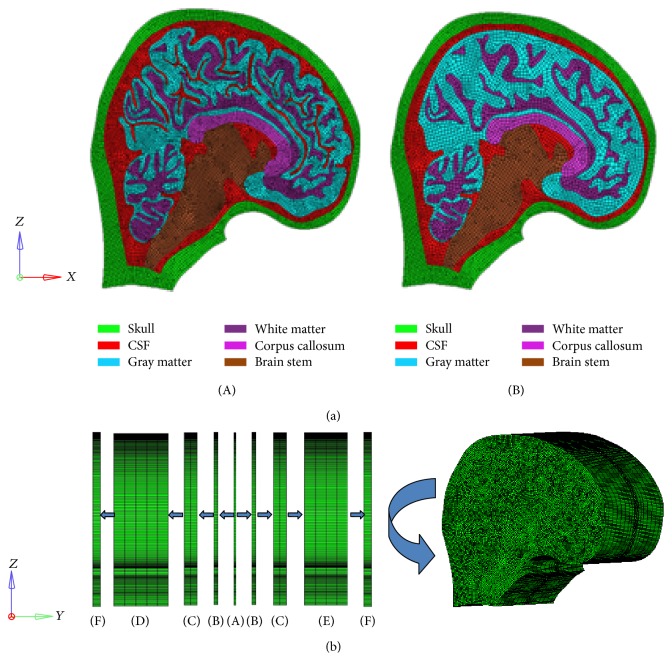
The development process of the models with and without gyri and sulci. (a) (A): the 2D model with gyri and sulci; (B): the 2D model without gyri and sulci. (b) Part (A) is extruded based on the 2D model with the thickness of 2 mm. The thickness of each layer from Part (B) to Parts (D) and (E) is 2 mm, 6 mm, and 10 mm, respectively, which aims to give Part (A) a support and avoid stress concentration in the simulation. Due to the support function to Part (A), a thickness gradient is selected in Part (B) to Parts (D) and (E). The thickness of Part (F) is 7 mm in order to depict the skull structure.

The geometry data of the human skull and brain in this study was obtained from an MRI scanning of a 170 cm tall healthy Chinese male from First Hospital of Jilin University. The MRI resolution was 256 by 256 pixels with pixel size of 0.997 mm. A parasagittal MRI image with an offset of 2 mm from midsagittal plane was selected as shown in Figure 1(a), and the selected parasagittal plane could represent detailed brain features more completely and clearly. The boundary characteristics of brain with gyri and sulci were described in CATIA V5 software based on the chosen MRI data, and the boundary lines of gyri and sulci were depicted according to the gray value boundary of MRI data. Then smoothing the boundary characteristics of the brain of the model with gyri and sulci got the model without gyri and sulci as shown in Figure 1(b)((A) and (B)). The model geometry data were imported into Hypermesh v11.0 and converted into FE mesh, so two two-dimensional models were built as shown in Figure 2(a) ((A) and (B), namely, one with gyri and sulci and the other without). The average mesh size was 2 mm. Two slice models were extruded from the two-dimensional models in *y* direction, respectively. Each slice model included five parts as shown in Figure 2(b)((A), (B), (C), (D) or (E), (F)). Part (A) was the main structure of the model, representing the parasagittal section of the head, and a thickness of 2 mm was assigned. Part (B) to Parts (D) and (E) were the support structures of Part (A), giving the same condition and location as the parasagittal section in the whole human head. A thickness gradient of 2 mm, 6 mm, and 10 mm was assigned from Part (B) to Parts (D) and (E), respectively. Part (F) depicted skull structure, and a thickness of 7 mm was assigned. The thickness of Part (A) was a thin layer, denoting the parasagittal section. The thickness of Part (B) has similar size of Part (A) to avoid stress concentration in the simulation, and a thickness gradient was assigned from Part (B) to Parts (D) and (E) in order to reduce element numbers of the whole model. The thickness of (F) was assigned according to the average skull thickness of actual head. The whole model mass reached human head average mass, that is, 4.75 kg [23]. It should be noted that, in Figure 2(b), Part (A) was not located in the center of the model because the slice was selected in the parasagittal position, rather than midsagittal plane. This caused the different layers of Part (D) and Part (E). The outermost layer of the model was solid which indicated skull structure. The constant stress solid element with one integration point was employed for all of the parts and the average element size was 2 mm. The slice model with gyri and sulci (Model 1) consisted of 239,169 nodes and 236,940 elements, and the model without gyri and sulci (Model 2) consisted of 139,629 nodes and 137,220 elements. Both models consisted of skull, CSF layer, and brain which further included cerebellum, brain stem, and corpus callosum. Gray matter and white matter were separated.

### 2.2. Material Properties

A large amount of brain tissue material properties has been reported in the literatures [20, 26]. As suggested in most of the publications, the brain tissue was modeled with viscoelastic behavior. The shear modulus of the viscoelastic brain *G*(*t*) has been given in the following expression:

(1) $$\begin{array}{c} G\left(\;t\;\right)=G_{\infty }+\left(\;G_{0}-G_{\infty }\;\right)e^{-\beta t}, \end{array}$$

where *G*_0_ is the short term shear modulus, *G*_*∞*_ is the long term shear modulus, and *β* is the decay factor. The gray matter was defined with a short term shear modulus of 10 kPa and a long term shear modulus of 2 kPa. The white matter was assumed to be 25% stiffer than the gray matter to account for their fibrous structure [23]. CSF was modeled as a viscoelastic solid that was assumed to be 10 times softer than the cerebral gray matter [20]. The material properties assigned to white matter were used in the corpus callosum. The brain stem was modeled with a short term shear modulus of 22.5 kPa and a long term shear modulus of 2.5 kPa. The skull was modeled as an elastic solid. Young's modulus (*E*), Poisson's ratio (*υ*), and density (*ρ*) for the skull were 15 GPa, 0.20, and 2,070 kg/m^3^, respectively. The values of the material constants are listed in Table 1.

### 2.3. Interface Conditions

In the head finite element model, how to model boundary conditions at the interfaces between different components in the head is critical. The models were built with sharing nodes between brain and CSF. Such constraint could prevent the formation of a gap between the CSF and cerebrum. This boundary condition is consistent with the models in the literature [9, 27–29], where the CSF has been modeled with a low stiffness and low shear modulus material to allow relative motion between the skull and the brain during head impact. The interfaces between other components in the head have been also implemented with node connection [18]. A surface-to-surface contact was set between the skull and the impactor, and a friction coefficient of 0.2 was used.

### 2.4. Element Size

In this paper, the element size of 2 mm was selected to mesh the slice models which can depict gyri and sulci and other brain features clearly. More detailed discussion of the influence of element size on simulation results was elaborated in the Discussion.

### 2.5. Impact Simulations

The analysis was performed with an explicit finite element code LS-Dyna (LSTC, 2006) that is commonly used for impact biomechanics and vehicle crash simulations. With this explicit FE code, the time step is computed at each step as a function of the smallest mesh size and its stiffness.

**Figure 3:**
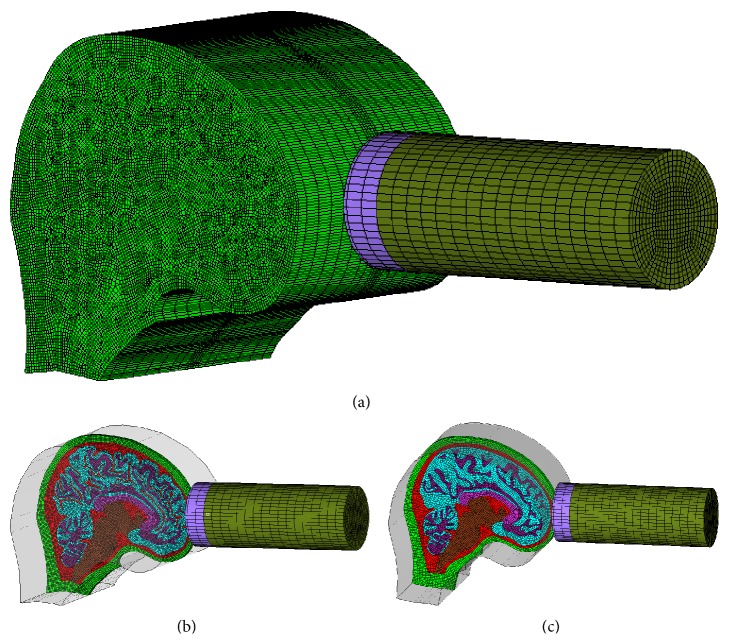
The loading conditions to simulate Nahum's experiment [24] ((a) enlarged view), the simulated scenario for Model 1 (b), and the simulated scenario for Model 2 (c).

Since the models had no neck, a free boundary was set at head-neck junction to simulate Nahum's impact experiment. It meant that there was no constraint at the head-neck junction. Ruan et al. and Willinger et al. [28, 30] pointed out that the neck had no significant effect on the head response under a short duration impact. To simulate Nahum's test, both models were impacted at the forehead by an impactor with an initial velocity of 3.6 m/s. The time duration of impact was 0.008 s. Since the material properties of the pad in Nahum's experiment were not published, a series of material properties were attempted to be assigned to the pad, and the material property which provided the consistent contact force between simulation and experiment data was selected and listed in Tables 2 and 3. The sketch of the loading conditions as well as Model 1 and Model 2 together with the impactor in the simulation is shown in Figure 3.

**Figure 4:**
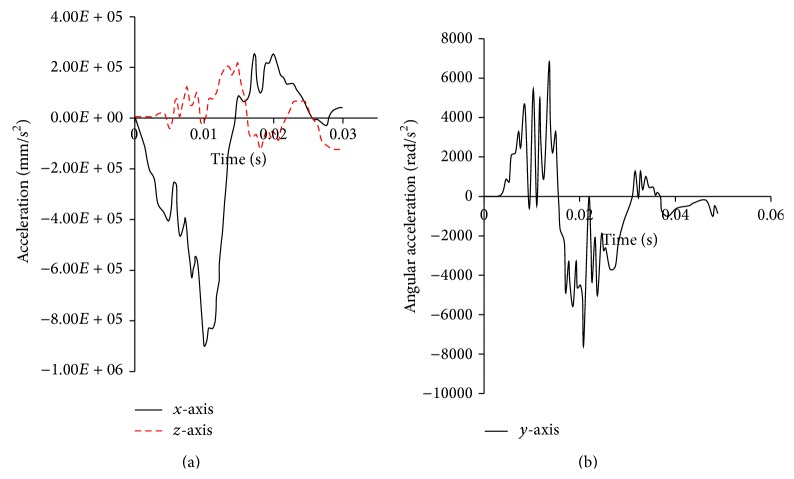
The accelerations applied to simulate the tests by Trosseille et al. [25]. (a) Translational acceleration along *x*-axis and *z*-axis. (b) Rotational acceleration along *y*-axis.

To simulate Trosseille's test, an acceleration curve was applied to replace the impact loading. The skull of each model was defined as rigid, and translational accelerations along *x*-axis and *z*-axis and a rotational acceleration along *y*-axis were loaded on the skull, which led to brain dynamic response as similar as experiment data. The acceleration curves applied are shown in Figure 4.

## 3. Results

### 3.1. FE Model Validation

**Figure 5:**
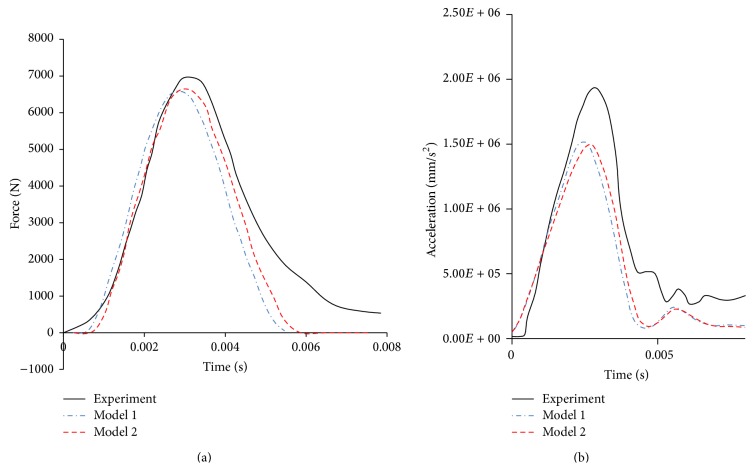
Comparison of model predicted and measured contact force and head acceleration. (a) Contact force-time history and (b) acceleration history.

**Figure 6:**
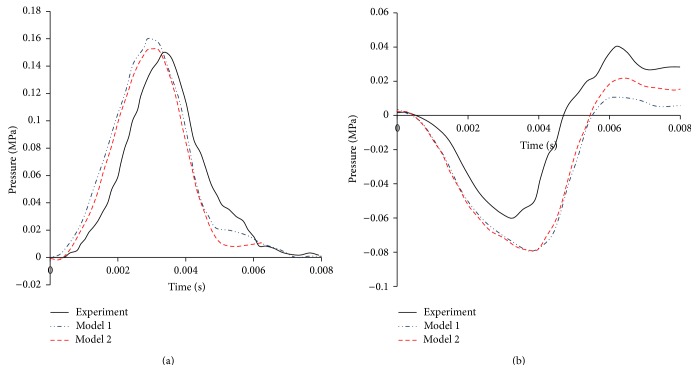
Pressure histories comparison between the numerical simulations and experiments by Nahum et al. (a) Coup site and (b) contrecoup site.

The model predicted contact forces and X-acceleration are compared with the cadaver tests by Nahum in Figures 5(a) and 5(b), respectively.  Figure 5(a) shows that the contact force curves in Model 1 and Model 2 are similar but both peak forces are lower than the test data. The discrepancy between simulation results and experiment data is within 10%, which indicates a reasonable agreement. The simulated accelerations at the CG in both models are compared with the test data in Figure 5(b). The trend can agree with the experimental data but the magnitude was underestimated and the relative error was about 32%. This may be due to differences in head mass and material properties between the current models and experimental cadaver samples. The coup and contrecoup pressure histories in the simulations based on both models and those measured in Nahum's test are plotted in Figures 6(a) and 6(b), respectively.  Figure 6(a) shows that Model 1 and Model 2 capture the experimental measured response reasonably well, with a small difference (less than 7%) in terms of coup pressure peak value. The negative pressures in the contrecoup region in both models have similar trends with the measured response. However, the peak negative pressures were overpredicted by 21% in both models. This may be due to simplification of the slice models and differences on contact condition and material properties between the current models and experimental samples.

### 3.2. Pressure

**Figure 7:**
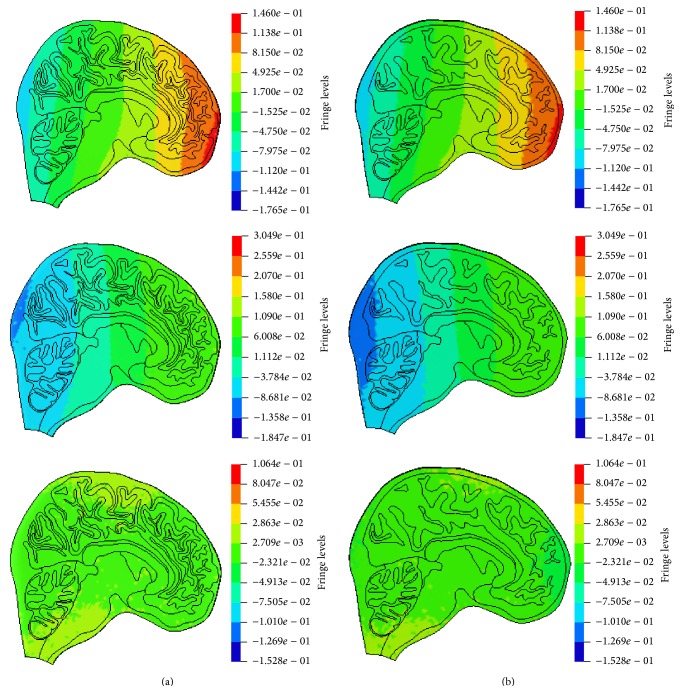
Pressure distributions (in MPa) predicted in the two models at the different time points. (a) Model 1 at *t* = 0.0024 s, 0.004 s, and 0.006 s and (b) Model 2 at *t* = 0.0024 s, 0.004 s, and 0.006 s.

Figures 6(a) and 6(b) show the pressures in coup and contrecoup sites for Model 1 and Model 2. It can be observed that general shape trend, magnitude, and duration of the pressure pulse in Model 1 agree well with those in Model 2. The predicted pressure distributions in Model 1 and Model 2 at three typical time points (*t* = 0.0024 s, 0.004 s, and 0.006 s) are shown in Figures 7(a) and 7(b), respectively. The pressure level in Model 1 is higher than that of Model 2 at all of the three time points.

### 3.3. Stress

**Figure 8:**
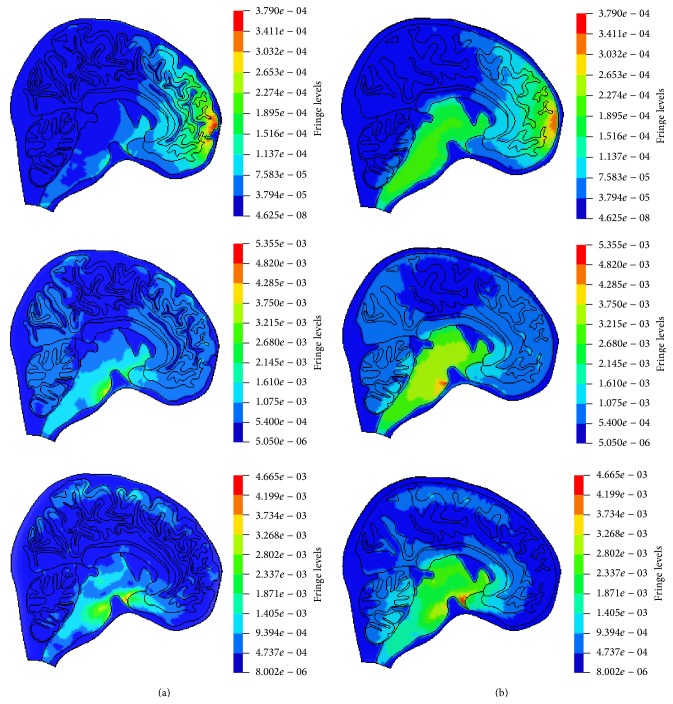
The model predicted equivalent stress distribution (in MPa) in both models at the different time points. (a) Model 1 at 0.0015, 0.0035, and 0.0065 s and (b) Model 2 at 0.0015, 0.0035, and 0.0065 s.

The von Mises stress distributions (in MPa) of Model 1 and Model 2 at 0.0015 s, 0.0035 s, and 0.0065 s in the simulations of Nahum's tests are shown in Figures 8(a) and 8(b), respectively. The stress concentration in Model 1 presents at the surface of brain between two gyri and the peak stresses are higher than that in Model 2.

**Figure 9:**
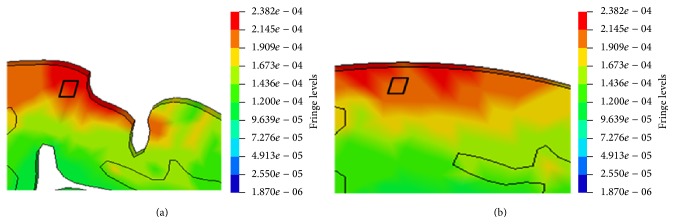
The stress distribution (in MPa) in the similar local fields of the cerebrums of Model 1 and Model 2. (a) Model 1 and (b) Model 2.

**Figure 10:**
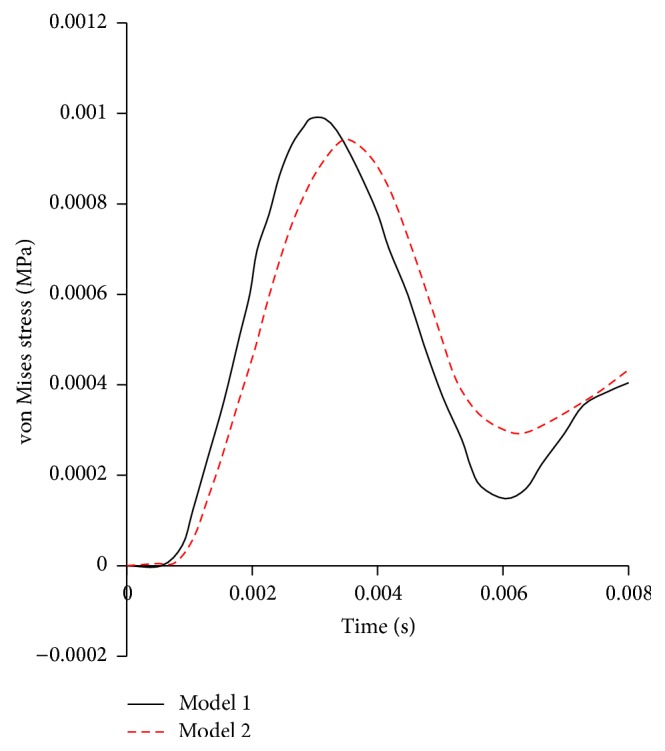
The von Mises stress comparison of local fields of the cerebrums for both models.

The influences of gyri and sulci on the distribution of equivalent stress at similar local regions of both models are shown in Figures 9(a) and 9(b), respectively. The comparison between Figures 9(a) and 9(b) shows that the maximum von Mises stress in Model 1 is higher than Model 2 and the stress concentration exists between gyri and sulci. The von Mises stress time histories taken from the same local fields of the cerebrums in both models (marked with black rectangle in Figure 9) are shown in Figure 10. The maximum von Mises stress of the cerebrums of Model 1 is slightly higher than that in the same field of Model 2.

### 3.4. Strain

**Figure 11:**
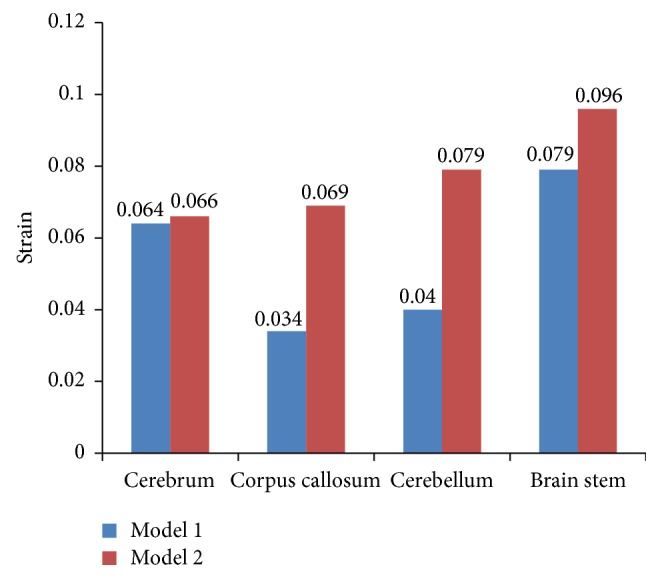
Comparison of maximum principle strains in different regions in both models.

The maximum principal strains in different regions of both models are listed in Figure 11. It is shown that the maximum principal strains in cerebrum, corpus callosum, cerebellum, and brain stem of Model l are lower than those in the same field in Model 2 by 3%, 51%, 38%, and 16%, respectively.

### 3.5. Dynamic Response

**Figure 12:**
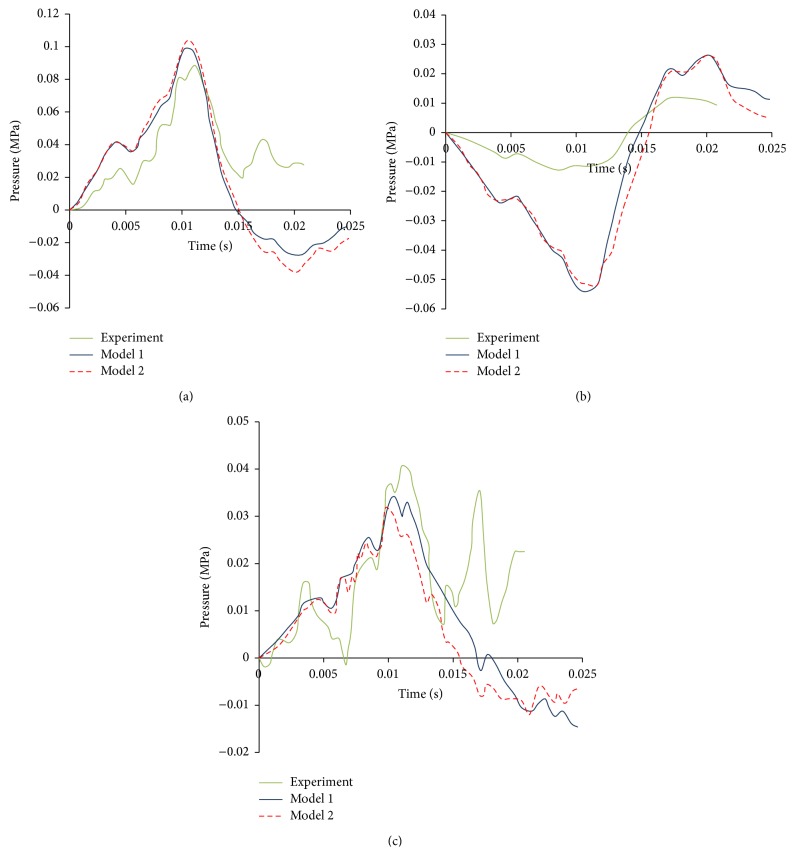
Model predicted pressure history compared with Trosseille's test in four different regions. (a) Coup site. (b) Contrecoup site. (c) Lateral ventricle.

The model predicted pressures are compared with Trosseille's test results in the coup, contrecoup, and lateral ventricle areas in Figures 12(a)–12(c), respectively.  Figure 12(a) shows that the coup pressures in the simulation agree fairly well with experimental data during 0 to 0.015 s. The difference in the curve shape starts after 0.015 s. The negative pressures were found in the simulation curves in Model 1 and Model 2 but not in the test. This is a major deficiency in these models.  Figure 12(b) shows that the countercoup pressures in the simulation are overestimated by both models compared to the data measured in the experiments. An explanation will be elaborated in the Discussion.  Figure 12(c) shows that lateral ventricle pressures in the simulation reasonably match the experimental data before 0.015 s. After 0.015 s, negative pressures were found in the simulation but not in the experiments.

Comparison of the pressure predictions of Model 1 and Model 2 in coup, contrecoup, and lateral ventricle sites indicates that the pressure histories of both models are similar in the magnitudes and time duration. This result reveals that the existence of gyri and sulci has little effect on the pressure caused by accelerations.

## 4. Discussion

### 4.1. Material Property

In this paper, two slice models were built to investigate the biomechanical response in a sagittal section during head impact. Unlike the other 2D FE models [22], present models were built by assigning different material properties to different parts of the head, similar to 3D FE head model reported in the literatures [18, 20, 21].

The brain material properties for the two-dimensional model, the three-dimensional model, and the current slice model are listed in Table 4. As shown in the table, *G*_0_ and *G*_*∞*_ in Kuijpers et al.'s paper [22] were 338 kPa and 169 kPa, respectively, which were 56 and 140 times higher than those in Mao et al.'s paper [23] where *G*_0_ and *G*_*∞*_ were 6 kPa and 1.2 kPa, respectively. The value of *K* was reduced by about 264 times in Kuijpers model than that in Mao's model. It can be seen that the material property values of the slice models were close to the three-dimensional model developed by Mao et al., and the material property of a two-dimensional model seems stiffer than the material property of a three-dimension model.

### 4.2. Stress

The comparison of the von Mises stress between Model 1 and Model 2 is made in Figures 8(a) and 8(b), respectively. It was found that stress concentrations took place at the edge of the brain and below two gyri in Model 1. This indicates that the presence of gyri and sulci leads to the higher local stress, which is highlighted in Figures 9(a) and 9(b). Gyri and sulci are generated as a result of the convolution of the cerebral cortex. The curvature of gyri and sulci enlarges the brain surface, and it is prone to cause stress concentration. Strich [31] made a comparison between the simulation results and clinically observed injury and found that there were some small cortical infarcts in brain injury at the bottom of gyri and sulci. This finding agrees with the present simulation result that stress concentrations exist between two gyri as shown in Figure 9(a).

### 4.3. Strain

**Figure 13:**
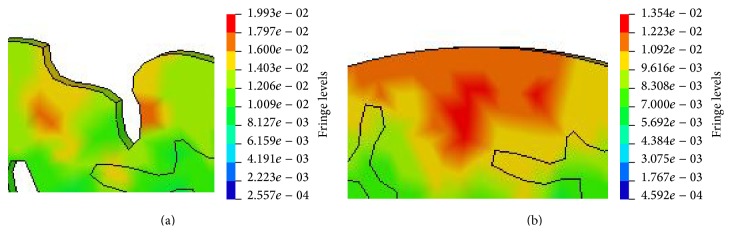
The strain distribution (in MPa) in the similar local fields taken from the cerebrums of Model 1 and Model 2. (a) Model 1 and (b) Model 2.

The comparison of maximum principal strain between Model 1 and Model 2 is made in Figure 11. The peak strain values in different regions of Model 1 were lower than the strains in Model 2, which was again caused by gyri and sulci. Due to the existence of gyri and sulci, the mechanical properties of Model 1 and Model 2 were different, which further resulted in the difference of maximum principal strain. The comparison in Figures 13(a) and 13(b) shows the higher strain is concentrated below gyri and sulci in Model 1. These alterations agreed with some physiological examination results which indicated that white matter deteriorations existed below gyri and sulci of the patients who suffered from head injuries [32].

### 4.4. Dynamic Response

In Figure 12(b), the contrecoup pressures of both models are much higher than the values in Trosseille's test. This trend can also be found in Turquier et al.'s work [32]. The discrepancy is related to loading condition. In Trosseille's experiment, an impactor was used to hit the head. The contact force generated caused deformation of the skull and then released the intracranial forces. This was neglected in the current models, since an acceleration curve was applied to replace the impact loading. Such setup may cause the discrepancy in the contrecoup pressure.

### 4.5. Gyri and Sulci

Most of the previous head models contained no gyri and sulci, so stress and strain predicted by these models may not reflect the actual responses below gyri and sulci. In this paper, the simulation results indicate that gyri and sulci would influence the stress and strain level and the areas of stress and strain concentrations during impact as shown in Figures 9(a) and 13(a), respectively. The local strain and stress could further affect the function of head after impact and would cause hematoma and edema in brain tissue when their values are high enough. Hence, gyri and sulci should be included in the FE head models to ensure more accurate injury assessments.

### 4.6. The Influence of Element Size on Simulation Results

In this paper, the element size of slice model (Part I) was 2 mm. In order to investigate the effect of element size on the simulation results, a refined mesh of Model 1 was built. Nahum's and Trosseille's experiments were simulated with two different mesh sizes and the simulation results are listed in Table 5. In the simulations of Nahum's tests, when the element size was reduced from 2 mm to 1 mm, the coup and countercoup pressure changed from 165 kPa and −85 kPa to 153 kPa and −89 kPa, respectively. The relative errors for coup and countercoup pressure are 7.2% and 6.7%, respectively. In the simulations of Trosseille's experiment, it can be found that the relative error of coup, countercoup, and lateral ventricle pressure is 5%, 7%, and 4%, respectively. When the element size was 2 mm, the simulation time for Nahum's experiment and Trosseille's test with 4 CPUs was 20 minutes and 36 minutes, respectively. Yet the simulation time became 2 hours 55 minutes and 7 hours 7 minutes with the mesh size reduced to 1 mm. This result suggests that larger element (2 mm) should be used in the current simulation in order to reduce the computation time with acceptable accuracy.

### 4.7. General Remarks

This study reveals the influence of gyri and sulci on the biomechanical response. In order to better illustrate the effect of gyri and sulci in the 3D sense, a more detailed and accurate three-dimensional FE model with gyri and sulci needs to be developed in the future work.

## 5. Conclusions

- The present finite element head models with and without gyri and sulci were validated with Nahum' experiment data. The coup and contrecoup pressures predicted in the simulations have a good agreement with the test data.
- The influence of gyri and sulci on the intracranial pressure was insignificant in the simulation of Nahum's and Trosseille's experiments. In the model with gyri and sulci, higher pressures occur at the edges of the brain tissue and below gyri and sulci.
- The model with gyri and sulci had a larger equivalent stress below gyri and sulci than the model without gyri and sulci in the same location.
- It is demonstrated that the strain distribution in the brain tissue was significantly changed due to gyri and sulci.

## Figures and Tables

**(a):** 

Part	Material law	*ρ* (kg/m^3^)	*K* (MPa)	*G*_0_ (kPa)	*G*_*∞*_ (kPa)	*β* (s^−1^)	References
White matter	Viscoelastic	1140	2190	12.5	2.5	80	[18, 19]
Gray matter	Viscoelastic	1140	2190	10	2.0	80	[18, 19]

Corpus callosum	Viscoelastic	1140	2190	12.5	2.5	80	[18, 19]

Brain stem	Viscoelastic	1140	2190	22.5	4.5	80	[18–20]

CSF	Viscoelastic	1040	1050	1.0	0.9	80	[18–20]

**(b):** 

Part	Material law	*ρ* (kg/m^3^)	*E* (MPa)	Poisson's ratio	References
Skull	Elastic	2070	15000	0.2	[19, 21]

**Table 2:** Material properties of the impactor.

Part	Material law	*ρ* (kg/m^3^)	*E* (MPa)	*G*_0_ (kPa)	*G*_*∞*_ (kPa)	*β* (s^−1^)	Poisson's ratio
Pad	Viscoelastic	80	2000	2300	5400	1.1	
Impactor	Rigid	7830	207000				0.3

**Table 3:** Dimensions of the impactor.

Part	Diameter (mm)	Length (mm)	Mass (kg)
Pad	72	22	7.14 × 10^−3^
Impactor	72	170	5.40

**Table 4:** The material properties comparison for 2D model, slice model, and 3D model.

Model	Brain tissue					
	Material law	*ρ* (kg/m^3^)	*K* (MPa)	*G*_0_ (kPa)	*G*_*∞*_ (kPa)	*β* (s^−1^)
2D model [22]	Viscoelastic	1040	8.3	338	169	50
Slice model	Viscoelastic	1140	2190	10	2	80
3D model [23]	Viscoelastic	1060	2190	6	1.2	80

**Table 5:** Comparison of simulation results and computational time using Model 1 with two different element sizes.

Simulation	Element size (mm)	Number of elements	MCP (kPa)	MCTP (kPa)	MLVP (kPa)	Computing time
Nahum experiment	1 mm	1,842,400	153	−89		2 hours 55 min
	2 mm	236,940	165	−83		20 min
			150	−60		
Trosseille experiment	1 mm	1,842,400	99	−54	28	7 hours 7 min
	2 mm	236,940	104	−58	29	36 min
			90	−15	39	

*Note*. MCP indicates maximum coup pressure, MCTP indicates maximum countercoup pressure, and MLVP indicates maximum lateral ventricle pressure.
